# The value of the continuous genotyping of multi-drug resistant tuberculosis over 20 years in Spain

**DOI:** 10.1038/s41598-020-77249-x

**Published:** 2020-11-24

**Authors:** María José Iglesias, Daniel Ibarz, Alberto Cebollada, Jéssica Comín, María Soledad Jiménez, María C. Vázquez, Sofía Samper, The Spanish Working Group on MDRTB, T. Cabezas, A. Reyes, I. Ruiz, P. García, M. D. López, L. Cardeñoso, I. Jesús de la Calle, P. Ruiz, J. C. Alados, J. Román, R. Villa-Real, J. Saavedra, C. Amores, P. Bermúdez, M. A. Sánchez, N. Montiel, S. Bernal, J. A. Lepe, N. Batista, E. Roldán, L. Torres, C. Navarro, P. Chocarro, M. J. Aldea, J. Viñuelas, M. A. Vitoria, J. J. Palacios, H. Villar, P. Prendes, M. Blanco, F. Vázquez, M. Telenti, I. Sánchez, L. Carbo, S. Escobar, A. Ramírez, C. Gallegos, M. C. Pérez, M. Lecuona, O. Díez, R. Copado, I. Campos, F. Cañas, C. Salas, C. Fernández, M. P. Roíz, I. Barba, E. Manrique, R. Carranza, A. Sánchez Maroto, A. González, E. Rodríguez, V. Martino, C. Sánchez, C. Martínez, P. Robles, E. Simarro, C. Romero, R. López, M. D. Blanco, T. Nebreda, J. Rodríguez, J. M. Fernández, E. Álvarez, M. L. Jaime, M. D. Tejero, A. Alberte, E. Oteda, G. Megías, C. Labayru, R. Ibáñez, A. Campos, P. Carrero, J. M. Villó, T. Sans, I. Pujol, X. Clivillé, J. B. Castellví, J. de Batlle, D. Mariscal, C. Prat, M. García, F. Alcalde, C. Gallén, G. Sauca, E. Cuchi, C. Alonso, F. Corcoy, G. Schmidt, M. T. Tortola, E. Garduño, J. J. Moreno, P. Hernández, I. Montes, J. Roman, P. Alonso, A. Rodríguez, L. Barbeyto, B. Fernández, D. Domínguez, R. Villanueva, I. Iglesias, F. J. Vasallo, J. Sevillano, A. Pascual, M. García, M. L. Pérez del Molino, V. Martino, E. Ugalde, R. Dopereiro, J. A. Cuadros, I. Pelayo, J. Cacho, R. Cogollos, M. Páez, S. Prieto, R. Fernández, P. López, D. Domingo, R. Millán, I. Bonilla, P. Merino, C. Toro, M. J. Ruiz, M. Menéndez, P. Romero, M. Tato, M. Simón, A. Urmeneta, A. Delgado, L. García, J. Cobos, J. Merino, E. Aznar, J. Piqueras, M. D. Navarro, J. M. Artero, A. Navascués, A. Gil, J. Leiva, L. Elorduy, E. Urra, P. Idígoras, E. Pérez-Trallero, A. Canut, J. L. Barrios, L. Michans, R. Ayarza, F. García, M. J. Unzaga, M. Navarro, N. Gonzalo, C. Martín, C. Martínez, A. Gimeno, M. Elia, P. López, S. Sabater, J. C. Rodríguez, M. Santos, M. Bosque, J. López, E. Tabernero, M. I. Galán

**Affiliations:** 1 Universidad de Zaragoza, Zaragoza, Spain; 2 CIBER de Enfermedades Respiratorias, Madrid, Spain; 3 Instituto Aragonés de Ciencias de La Salud, Zaragoza, Spain; 4 Laboratorio de Investigación Molecular-UIT, IIS Aragón, Instituto Aragonés de Ciencias de La Salud, Hospital Universitario Miguel Servet, Pº Isabel la Católica 1-3, planta calle, CP 50009 Zaragoza, Aragón Spain; 5 Centro Nacional de Microbiología, Instituto de Salud Carlos III, Madrid, Spain; 6 Ministerio de Sanidad, Madrid, Spain; 7 H. Poniente, El Ejido, Almería, Spain; 8 H. Torrecárdenas, Almería, Spain; 9 H. Punta de Europa, Algeciras, Spain; 10 H. Univ. Puerta del Mar, Cádiz, Spain; 11 H. de Jerez de la Frontera, Cádiz, Spain; 12 H. Comarcal Puerto Real, Cádiz, Spain; 13 Hosp. Univ. de Puerto Real, Cadiz, Spain; 14 H. Reina Sofía, Córdoba, Spain; 15 H. Virgen de la Nieves, Granada, Spain; 16 Hosp. Univ. San Cecilio, Granada, Spain; 17 H. San Juan de la Cruz, Úbeda, Jaén, Spain; 18 H. Juan Ramón Jiménez, Huelva, Spain; 19 H. San Agustín, Linares, Spain; 20 Complejo H. Carlos Haya, Málaga, Spain; 21 H. Ntra. Sra. de la Victoria, Málaga, Spain; 22 H. Costa del Sol, Marbella, Spain; 23 H. Ntra. Sra. De Valme, Sevilla, Spain; 24 H. Virgen del Rocío, Seville, Spain; 25 H. Virgen de la Macarena, Sevilla, Spain; 26 H. La Merced, Osuna, Sevilla, Spain; 27 H. San Jorge, Huesca, Spain; 28 H. de Alcañiz, Teruel, Spain; 29 H. Obispo Polanco, Teruel, Spain; 30 H. Royo Villanova, Zaragoza, Spain; 31 H. Univ. Miguel Servet, Zaragoza, Spain; 32 H. Univ. Lozano Blesa, Zaragoza, Spain; 33 H. San Agustín, Avilés, Spain; 34 H. de Cabueñes, Gijón, Spain; 35 H. Monte Naranco, Oviedo, Spain; 36 H. Gral. de Asturias, Oviedo, Spain; 37 Instituto Nacional de Silicosis, Oviedo, Spain; 38 H. Verge del Toro, Mahon, Spain; 39 Policlínica Miramar, Palma de Mallorca, Spain; 40 H. Univ. Son Espases, Palma de Mallorca, Spain; 41 H. Joan March, Bunyola, Mallorca Spain; 42 H. Son Llatzer, Palma de Mallorca, Spain; 43 H. Univ. de Canarias, La Laguna, Spain; 44 H. Ntra. Sra. Candelaria, Sta. Cruz de Tenerife, Spain; 45 H. Gral. de Lanzarote, Madrid, Spain; 46 H. Doctor Negrín, Las Palmas, Spain; 47 H. Insular, Las Palmas, Spain; 48 H. Sta. Cruz, Liencres, Cantabria Spain; 49 H. Marqués de Valdecilla, Santander, Spain; 50 H. Sierrallana, Torrelavega, Spain; 51 H. Univ. de Ciudad Real, Ciudad Real, Spain; 52 H. Gutiérrez Ortega, Valdepeñas, Spain; 53 H. General La Mancha Centro, Alcázar de San Juan, Spain; 54 Hosp. Virgen de Altagracia, Manzanares, Spain; 55 H. Gral. de Guadalajara, Madrid, Spain; 56 H. Virgen de la Salud, Toledo, Spain; 57 H. Ntra. Sra. del Prado, Talavera de la Reina, Spain; 58 H. Virgen de la Luz, Cuenca, Spain; 59 H. Gral. de Albacete, Madrid, Spain; 60 H. de Hellín, Albacete, Spain; 61 H. del Bierzo, Ponferrada, Spain; 62 H. Monte San Isidro, León, Spain; 63 Complejo H. de León, León, Spain; 64 H. Virgen de la Concha, Zamora, Spain; 65 H. Clínico Univ. de Salamanca, Madrid, Spain; 66 H. Gral. Río Carrión, Palencia, Spain; 67 H. San Telmo, Palencia, Spain; 68 H. Clínico Univ. de Valladolid, Madrid, Spain; 69 H. Río Hortega, Valladolid, Spain; 70 H. Gral. Yagüe, Burgos, Spain; 71 H. Univ. de Burgos, Burgos, Spain; 72 H. Ntra. Sra. de Sonsoles, Ávila, Spain; 73 H. de Soria, Madrid, Spain; 74 H. de Segovia, Madrid, Spain; 75 H. Valls, Tarragona, Spain; 76 H. Mora de Ebro, Tarragona, Spain; 77 H. Sant Joan de Reus, Tarragona, Spain; 78 H. San Pau i Santa Tecla, Tarragona, Spain; 79 H. Doctor Trueta, Girona, Spain; 80 H. Parc Taulí, Sabadell, Barcelona, Spain; 81 H. Universitari Germans Trias i Pujol, Badalona, Barcelona, Spain; 82 H. Arnau de Vilanova, Lérida, Spain; 83 H. Univ. de Bellvitge, Hospitalet de Llobregat, Barcelona, Spain; 84 H. San Jaume, Calella, Barcelona, Spain; 85 Consorci Sanitari de Mataró, Barcelona, Spain; 86 H. Mutua de Terrassa, Barcelona, Spain; 87 H. de la Cruz Roja, Hospitalet de Llobregat, Barcelona, Spain; 88 H. San Camilo, San Pere de Ribes, Barcelona, Spain; 89 H. de Terrasa, Barcelona, Spain; 90 Hospital Universitari Vall d’Hebron, Barcelona, Spain; 91 H. Univ. Infanta Cristina, Badajoz, Spain; 92 H. de Mérida, Badajoz, Spain; 93 H. Ciudad de Coria, Cáceres, Spain; 94 H. Virgen del Puerto, Plasencia, Spain; 95 H. de Monforte, Lugo, Spain; 96 H. Xeral de Calde, Lugo, Spain; 97 H. Cristal Piñor, Orense, Spain; 98 H. Santa Maria Nai, Orense, Spain; 99 H. Arquitecto Marcide, Ferrol, Spain; 100 H. Juan Canalejo, Coruña, Spain; 101 H. Xeral-Cíes, Vigo, Spain; 102 H. Meixoeiro, Vigo, Spain; 103 Clínica Povisa, Vigo, Spain; 104 H. Montecelo, Pontevedra, Spain; 105 H. Provincial, Pontevedra, Spain; 106 Complejo Univ. de Santiago de Compostela, Madrid, Spain; 107 H. de Conxo, Santiago de Compostela, Spain; 108 H. San Pedro, La Rioja, Spain; 109 H. Gral. de la Rioja, Logroño, Spain; 110 H. Príncipes de Asturias, Alcalá de Henares, Spain; 111 H. Fuenfría, Cercedilla, Spain; 112 H. Univ. de Getafe, Madrid, Spain; 113 H. de Móstoles, Madrid, Spain; 114 H. Severo Ochoa, Leganés, Madrid, Spain; 115 H. de Fuenlabrada, Madrid, Spain; 116 Fundación Jiménez Díaz, Madrid, Spain; 117 H. Doce de Octubre, Madrid, Spain; 118 H. de la Princesa, Madrid, Spain; 119 H. Puerta de Hierro, Madrid, Spain; 120 H. San Carlos, Madrid, Spain; 121 H. Carlos III, Madrid, Spain; 122 H. Gregorio Marañón, Madrid, Spain; 123 H. Infantil Univ. Niño Jesús, Madrid, Spain; 124 H. La Paz, Madrid, Spain; 125 H. Ramón y Cajal, Madrid, Spain; 126 H. Central de la Defensa Gómez Ulla, Madrid, Spain; 127 H. Enfermedades del Tórax, Cantoblanco, Madrid, Spain; 128 H. Univ. Fundación de Alcorcón, Alcorcón, Spain; 129 Hosp. de El Escorial, Madrid, Spain; 130 MEGALAB, Madrid, Spain; 131 UNILABS, Madrid, Spain; 132 Laboratorio BR Salud. Laboratorio Central de Madrid, San Sebastián de los Reyes, Spain; 133 H. Sta. María del Rosell, Cartagena, Spain; 134 H. Morales Messeguer, Murcia, Spain; 135 H. Reina Sofía, Murcia, Spain; 136 Complejo hospitalario de Navarra, Pamplona, Spain; 137 Clínica Universitaria de Navarra, Pamplona, Spain; 138 H. San Eloy, Baracaldo, Spain; 139 H. de Cruces, Baracaldo, Spain; 140 Complejo H. Donostia, San Sebastián, Spain; 141 H. Santiago Apóstol, Vitoria, Spain; 142 H. Txagorritxu, Vitoria, Spain; 143 H. de Galdakao, Vizcaya, Spain; 144 H. Sta. Marina, Bilbao, Spain; 145 H. de Basurto, Bilbao, Spain; 146 H. Vega Baja, Orihuela, Alicante, Spain; 147 H. Sant Joan, Alicante, Spain; 148 H. de Villajoyosa, Alicante, Spain; 149 H. Gral. Univ, Alicante, Spain; 150 H. Virgen de la Salud, Elda, Spain; 151 H. Verge dels Lliris, Alcoy, Spain; 152 H. Gral.de Castellón, Madrid, Spain; 153 H. Gral. de Elche, Madrid, Spain; 154 H. la Fe, Valencia, Spain; 155 H. Arnau de Vilanova, Valencia, Spain; 156 H. de la Cruz Roja, Ceuta, Spain; 157 H. de Melilla, Melilla, Spain

**Keywords:** Tuberculosis, Infectious diseases, Respiratory tract diseases, Phylogenetics, Clinical microbiology, Microbial genetics, Policy and public health in microbiology, Health care, Risk factors

## Abstract

Molecular epidemiology of circulating clinical isolates is crucial to improve prevention strategies. The Spanish Working Group on multidrug resistant tuberculosis (MDR-TB) is a network that monitors the MDR-TB isolates in Spain since 1998. The aim of this study was to present the study of the MDR-TB and extensively drug-resistant tuberculosis (XDR-TB) patterns in Spain using the different recommended genotyping methods over time by a national coordinated system. Based on the proposed genotyping methods in the European Union until 2018, the preservation of one method, MIRU-VNTR, applied to selected clustered strains permitted to maintain our study open for 20 years. The distribution of demographic, clinical and epidemiological characteristics of clustered and non-clustered cases of MDR/XDR tuberculosis with proportion differences as assessed by Pearson’s chi-squared or Fisher’s exact test was compared. The differences in the quantitative variables using the Student's-t test and the Mann–Whitney U test were evaluated. The results obtained showed a total of 48.4% of the cases grouped in 77 clusters. Younger age groups, having a known TB case contact (10.2% vs 4.7%) and XDR-TB (16.5% *vs* 1.8%) were significantly associated with clustering. The largest cluster corresponded to a *Mycobacterium bovis* strain mainly spread during the nineties. A total of 68.4% of the clusters detected were distributed among the different Spanish regions and six clusters involving 104 cases were grouped in 17 and 18 years. Comparison of the genotypes obtained with those European genotypes included in The European Surveillance System (TESSy) showed that 87 cases had become part of 20 European clusters. The continuity of MDR strain genotyping in time has offered a widespread picture of the situation that allows better management of this public health problem. It also shows the advantage of maintaining one genotyping method over time, which allowed the comparison between ancient, present and future samples.

## Introduction

Although global TB incidence and mortality rates have fallen in recent years, the number of confirmed multidrug-resistant TB (MDR-TB) cases, defined as resistant to at least isoniazid (INH) and rifampicin (RIF), has almost doubled globally over the past 5 years^1^. The high number of cases in some countries remains constant threat^2^. Spain has a low TB incidence rate of 9.4/100 000 population as reported in 2017^2^. Nevertheless, in 1998 the MDR condition was partially known. In January 1998 a network to monitor the spread of MDR-TB based on genotyping was set up to establish an early warning system^3^. Previous monitoring studies of this network conducted at 3 and 11 years allowed us to attain an understanding of the MDR-TB situation and spread of disease in Spain^4,5^. In addition to the classical genotypes, MDR-TB genotyping endorsed the use of resistance-associated mutations to INH and RIF which increased the discriminatory power^5^.

The knowledge on *Mycobacterium tuberculosis* genome has led to the search for the best genotyping method in molecular epidemiology*.* In the 1990’s, the Restriction Fragment Length Polymorphism (RFLP) based on IS*6110* was recognised as gold standard, and was the selected method for the surveillance in Europe by EuroTb^6^. The European Centre for Disease Prevention and Control (ECDC) proposed the mycobacterial interspersed repetitive unit-variable number of tandem repeat (MIRU-VNTR) since 2009 in the European Union^7^, and collected data in The European Surveillance System (TESSy). Until 2015, the Spanish network, contributed with 406 isolates being the second country in number included^8^. In 2019, ECDC proposed the use of whole genome sequencing (WGS) in surveillance and outbreak investigations for some priority pathogens^2^.

The present work reflects the MDR-TB situation in Spain until 2017, since the creation of the Spanish network on drug-resistant TB in 1998. It shows the results of the uninterrupted monitoring of circulating MDR-TB strains using the different techniques recommended by the Scientific Community, prior to the incorporation of WGS. It presents the MDR-TB patterns in Spain and their distribution around the country. In addition, the social, epidemiological and clinical characteristics of the patients were related with clustering to get a better understanding of the characteristics of the MDR/XDR TB population and to clarify the circulation routes of the disease in Spain.

## Methods

The Spanish Working Group network on MDR-TB integrates laboratories distributed across the different Spanish regions. These laboratories provided inactivated MDR isolates of *M. tuberculosis* as well as microbiological information to our Mycobacterial Genetics Group laboratory (located at the University of Zaragoza) to detect, analyse and notify the outbreaks as foreseen in the plan for the prevention and control of tuberculosis in Spain. The samples were encrypted by a technician with a code named "NSTRAIN", which included three capital letters ESC, and nine digits, the first four digits corresponded to the year of isolation of the strain, and the last five were given in the order in which the samples were received. The collected information included the hospital and the region where the isolations came from, susceptibility test results, and age, gender, country of birth, previous treatment and other major risk factors for TB of the cases^3^. The report of the genotyping results was sent back to the laboratories of origin and when similar genotypes involved different regions a report was sent to the National Centre for Epidemiology (CNE, abbreviation in Spanish). Since 2009, the genotypes obtained have been incorporated in TESSy^7^.

### Genotyping

**Figure 1:**
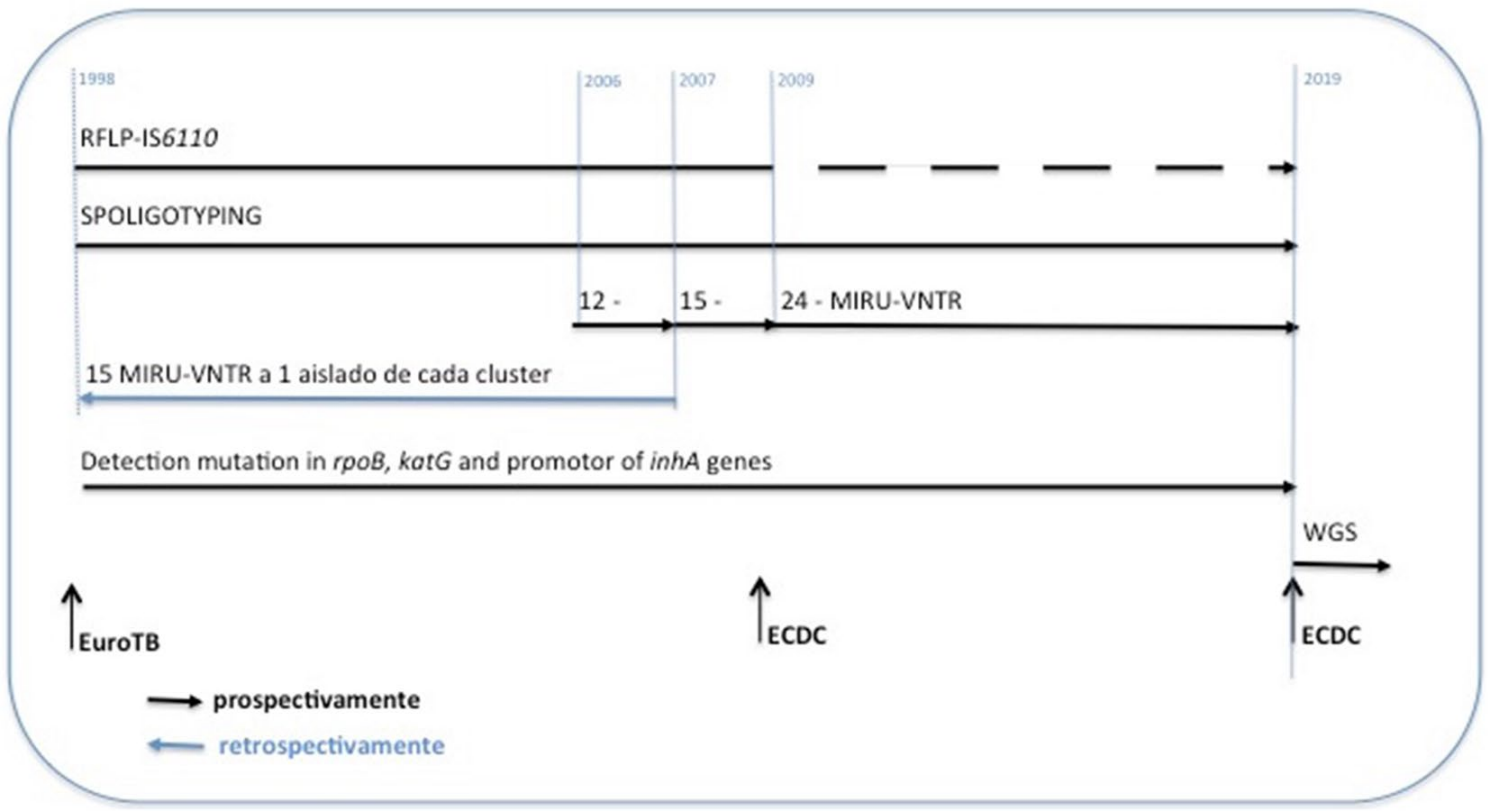
Diverse methods used along the study. From 1998 to 2009, IS*6110*-RFLP was the genotyping applied method. Standard 12-MIRU-VNTR was introduced in 2003, 15-MIRU-VNTR in 2006 and 24-loci MIRU-VNTR typing in 2009. Complementary, one isolate representative of each IS*6110*-RFLP cluster was genotyped by the 24-MIRU-VNTR. Spoligotyping and mutations in *katG* gene and promoter region of *inhA* gene were performed on all isolates of the study. Spoligo-International-Type (SIT) labels were assigned.

All inactivated MDR/XDR isolates received, during the period from January 1998 to December 2017, were genotyped. Only the first isolate per case was included. Those samples with not enough DNA to obtain a genotype were excluded. The proposed molecular standard methods used for genotyping, using IS*6110*-RFLP, MIRU-VNTR and Spoligotyping have been adapted and combined to permit the comparison among the genotypes along the different periods of the study (Fig. 1). The Spoligo-International-Type (SIT) labels were assigned^9^. The target genes for RIF and INH were analysed. The genotypes of the MDR cases based on 24-MIRU-VNTR were incorporated in TESSy^7^.

### Cluster analysis

In our study, isolates were considered in cluster if they carried identical mutations in *rpoB*, *katG* and/or *inhA* genes showed: (1) identical IS*6110*-RFLP pattern, and the same Spoligotype in case they had less than 5 IS*6110*; (2) identical 15-MIRU-VNTR profiles; (3) identical 12-MIRU-VNTR and spoligotype profiles. The genotypes were stored in Bionumerics 7.6v software (Applied Maths). Clustered cases were assumed to be epidemiologically related^5^. The index case was considered the first case detected in each cluster. The clusters were named receiving a correlative number preceded by “*tb”* for *M. tuberculosis* or “*bv”* for *M. bovis*. Exceptions were made with clusters involving Beijing strains, which were named *Beijing* followed by *pattern* and a consecutive number, and with those clusters considered evolved which maintained the original name followed by a letter.

### Statistical analysis

We compared the distribution of the characteristics of clustered and non-clustered cases with proportion differences as assessed by Pearson’s chi-squared or Fisher’s exact test when the cell count was less than five. We evaluated the differences in the distribution of the quantitative variables studied using the Student's-t test in the case of normal variables, or the Mann–Whitney U test in the case of non-parametric variables. We used Odds Ratio (OR) and 95% confidence intervals (CIs) to assess the association of variables with clustering. A p value of less than 0.05 was considered as statistically significant. All analyses were executed with R software (version 3.5.1).

### Ethics declarations

The planning conducted of the study was approved by the Comité de Ética de la Investigación de la Comunidad Autónoma de Aragón (CEICA), Spain, CI.PI18/068. The protocol followed is in line with the Declaration of Helsinki, as revised in 2013. Once received the bacterial isolate, it was coded (NSTRAIN). The information about the cases were sent by fax and was anonymised keeping only the code given to track the analysis of the clinical characteristics, to follow the Helsinki ethical principles for medical research involving human data. Comité de Ética de la Investigación de la Comunidad Autónoma de Aragón (CEICA) waived need for informed consent (CI.PI18/068).

## Results

### Analysis of the isolates by clustering

**Figure 2:**
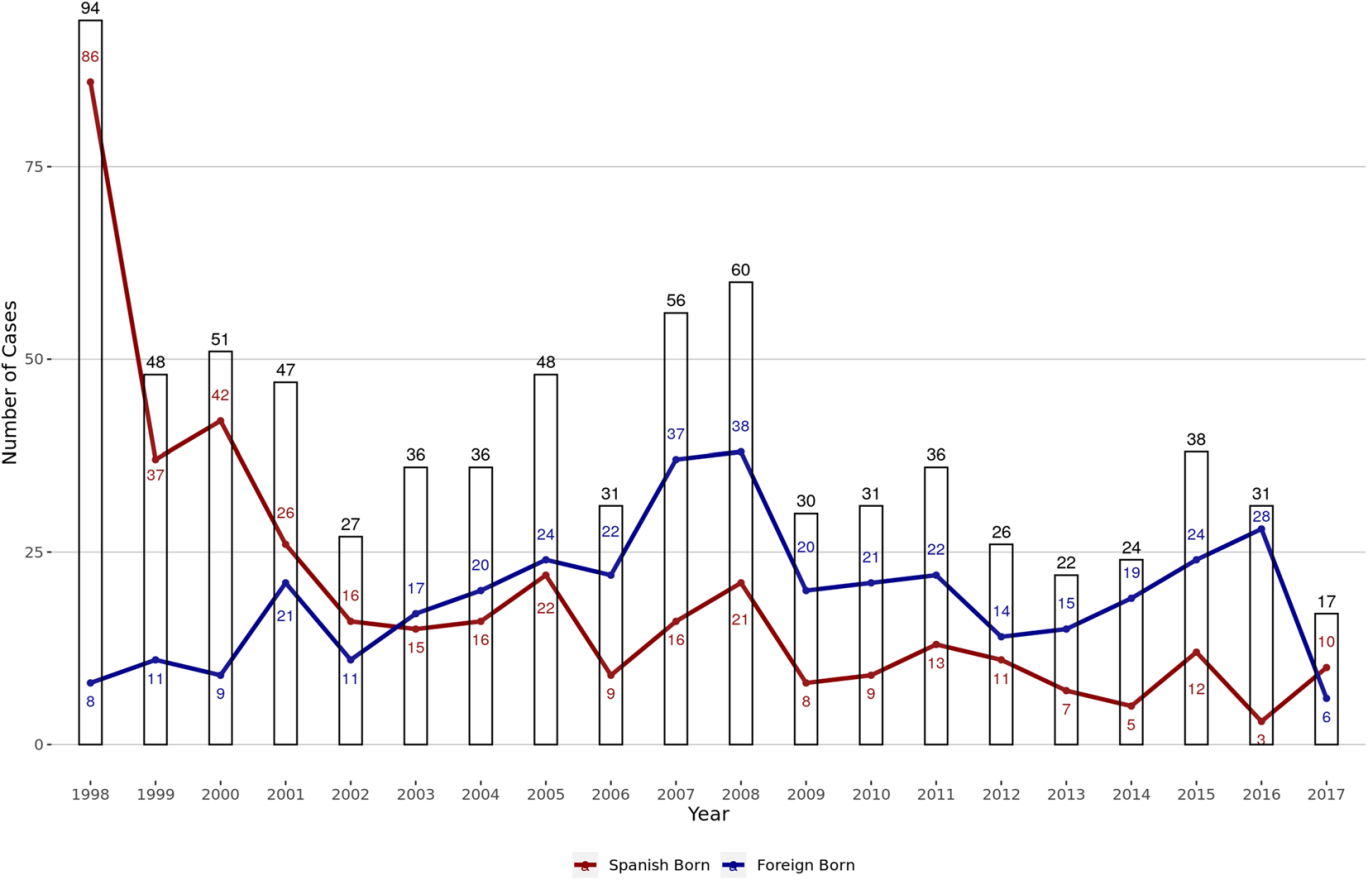
Distribution based on the origin of the cases along the 20 years of the study. The red line represents the native-born cases, blue line shows foreign-born cases and the bars represent the total number of cases.

The MDR/XDR *M. tuberculosis* complex isolates received were genotyped and further analysed. A total of 926 inactivated isolates from 834 patients were received during the study period from 1998 through 2017. In total, 137 samples were excluded due to laboratory cross-contaminations (n = 3), insufficient sample amount to obtain a genotype (n = 42), as well as multiple isolates from the same patient (n = 92). Finally, the studied population included 789 samples, corresponding to a similar number of patients. The number of isolates included per year is depicted in Fig. 2.

**Figure 3:**
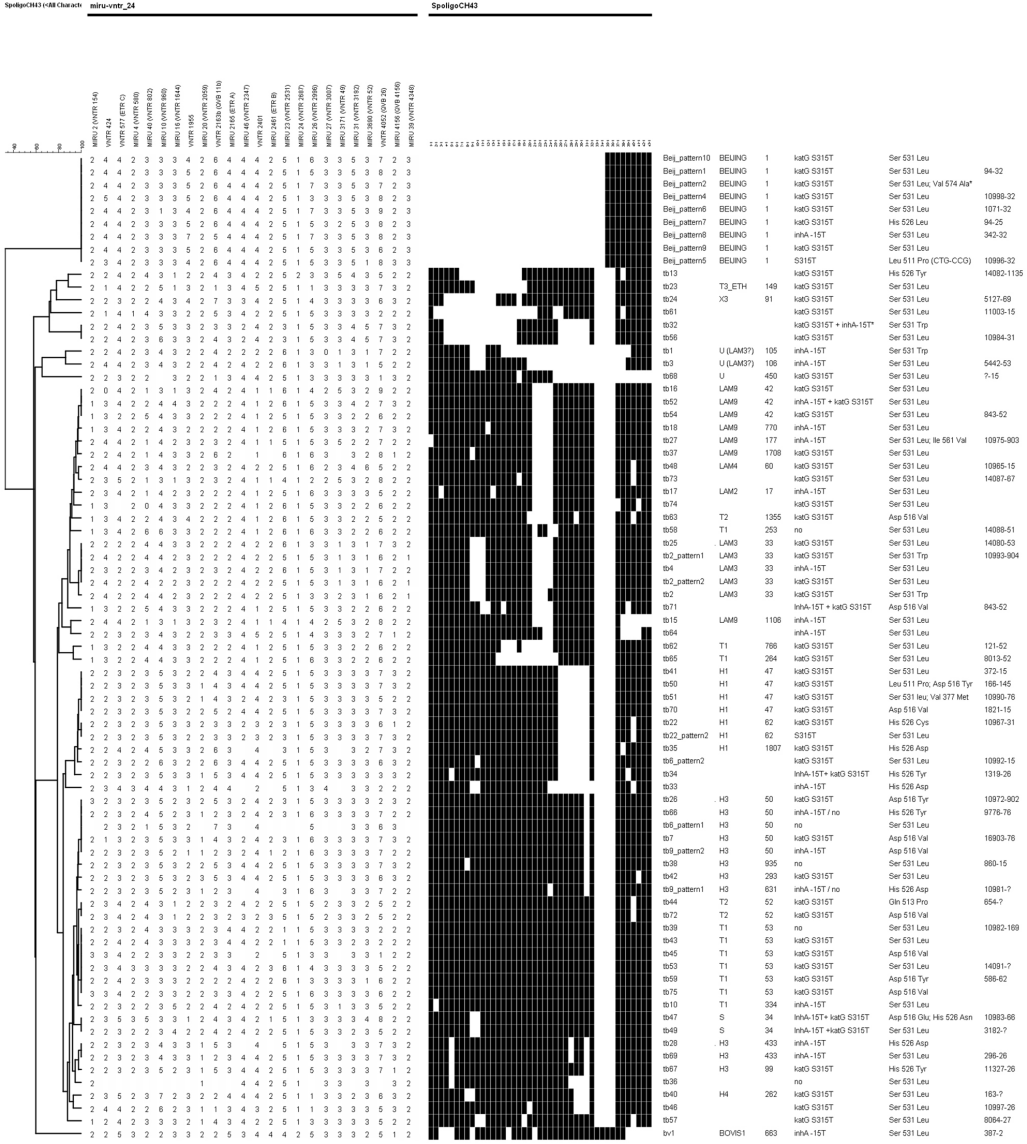
Spoligotyping based dendrogram of the 77 MDR *M. tuberculosis* cluster patterns from Spain. SIT, Spoligo-International Type. Mycobacterial interspersed repetitive-unit variable-number tandem-repeat (MIRU-VNTR) pattern of each cluster, mutations associated to resistance to isoniazid and rifampicine and the cluster name in TESSy are given.

**Figure 4:**
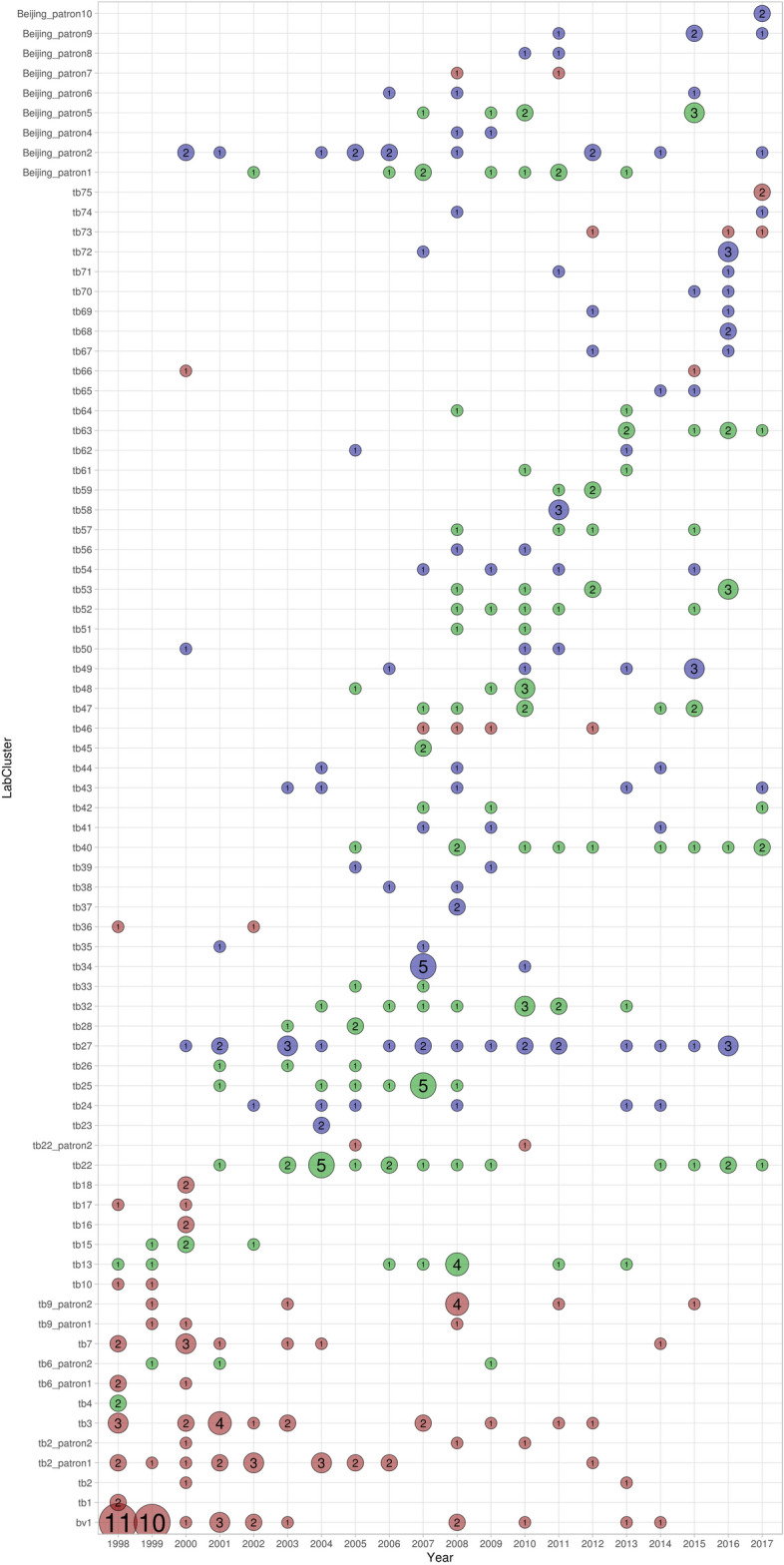
Distribution of the cases in clusters along the period studied. Red circles represent the native clusters, blue circles show foreign clusters and green circles show mixed clusters. The size of the circles is proportional to the number of the included isolates.

Among the 789 isolates, 48.4% grouped into 77 clusters (2–33 cases per cluster) (Fig. 3). According to their spoligotipe, 51 of the clusters belonged to Lineage (L) 4, 19 of which were L4.1.2 (Haarlem family), 15 were L4.3 (LAM family), two were L4.4.1 (S family), one was L4.1.1 (X family) and 14 clusters were assigned as L4 as T ill-defined family Nine of the clusters belonged to lineage 2 and one cluster belonged to Animal linage. Thirteen genotypes had no Spoligotype assigned and one was unknown. There was great variability in how the size and time-frame of clusters increased (Fig. 4). The persistence of clusters ranged between 1 and 18 years. For instance, cluster *tb34,* onset abruptly with five cases in the same year followed by a new case three years later; but others, as *tb40* cluster with 11 cases, occurred over 13 years with a maximum of two cases per year (Fig. 4). Among the 420 isolates with susceptibility results reported, 45 presented a XDR pattern (10.2%), three presented a unique genotype while 42 grouped in different clusters: *bv1* included 33 cases, meanwhile the other 9 were distributed among clusters: *tb26* (3 cases), *Beijing-pattern2* (2 cases), and *tb46*, *tb50*, *Beijing-pattern9* and *Beijing-pattern10* (1 case in each cluster).

### MDR/XDR TB population characteristics

The social, epidemiological and clinical data of the patients were revised in order to get a better understanding of the characteristics of the MDR/XDR TB population in Spain. According to the demographic characteristics, the gender was known for 95.9% (n = 757) of the cases, the gender-ratio M/F was 1.9:1. The age was available for 76.5% (n = 604) with a non-homogenous distribution, with the highest proportion of diagnoses occurring in individuals aged 15–34 years (41.5%). The overall mean age was 39.76 years (range 0–92 years), 42.70 (16.90 sd) years for male and 33.61 (16.49 sd) years for female (p < 0.001). Information about the country of origin of the patient was available for 97.6% (n = 770); 49.6% (n = 382) were Spanish-born and 50.4% (n = 388) were foreign-born, from 34 different countries. The majority coming from Europe Region (40.1%), followed by the Americas (34.3%), Eastern Mediterranean (10.1%), the African (9.4%), the South-East Asian (3.2%) and Western Pacific Region (0,4%).

With respect to the clinical characteristics, 90.5% presented pulmonary or pulmonary/extra-pulmonary TB, with 70.5% giving a positive sputum smear. Previous TB and TB contact was related by the patient in 55.2% and 14.7% of the cases, respectively. HIV status, intravenous drug user or alcohol abuse was a condition present in 20.7%, 13.1% and 17.7% respectively. The drug susceptibility of the 789 MDR-TB isolates showed that 525 presented any additional resistance and 45 (5.7%) were XDR, as they presented resistance to any fluoroquinolone and any of the three injectable aminoglycosides.

### Analysis of the sociodemographic and clinical characteristic of the patients by clustering

Cluster analysis by patients’ demographic characteristics was carried out to find existent correlations (Table 1). Among the male patients, 47.4% were clustered, versus 50.2% among the female patients. Clustering by age groups showed significant differences. Young patients from 0 to 14 years old were more in cluster (65%) whereas the group aged older than 55 years presented more unique patterns (67.5%). Foreign-born cases were significantly associated with clustering, OR = 1.34 (95% CI 1.01–1.78). The odds of belonging to a cluster was OR = 2.73 higher for patients with no previous tuberculosis (95% CI 1.89–3.97) and having a TB contact increased the likelihood of belonging to a cluster by OR = 2.71 (95% CI 1.52–4.98) and finally, XDR was significantly associated with clustering. Other demographic, clinical and microbiological characteristics analysed were not significant (Table 1).

**Table 1:** Sociodemographic and clinical characteristic of cases according to clustered and non-clustered tuberculosis cases.

	Cluster n = 382 (48.42%)	Unique n = 407 (51.58%)	Unadjusted odds ratio (95% CI)	P value
**Gender**				
Male	236 (47.39%)	262 (52.61%)	Ref	Ref
Female	130 (50.19%)	129 (49.81%)	1.12 [0.83;1.51]	0.465
**Age group**				
0–14	13 (65.00%)	7 (35.00%)	Ref	Ref
15–34	138 (54.98%)	113 (45.02%)	0.67 [0.24;1.70]	0.400
35–54	103 (47.03%)	116 (52.97%)	0.48 [0.17;1.24]	0.133
55 +	37 (32.46%)	77 (67.54%)	0.26 [0.09;0.71]	0.008
**Born origin**				
Spanish-Born	171 (44.76%)	211 (55.24%)	Ref	Ref
Foreign-Born	202 (52.06%)	186 (47.94%)	1.34 [1.01;1.78]	0.043
**Type of TB**				
Pulmonary or pulmonary/extra-pulmonary	266 (49.17%)	275 (50.83%)	Ref	Ref
Only extra-pulmonary	25 (43.86%)	32 (56.14%)	0.81 [0.46;1.40]	0.451
**Sputum smear**				
Positive	185 (50.68%)	180 (49.32%)	Ref	Ref
Negative	75 (49.02%)	78 (50.98%)	0.94 [0.64;1.37]	0.731
**Previous TB**				
Yes	97 (36.47%)	169 (63.53%)	Ref	Ref
No	132 (61.11%)	84 (38.89%)	2.73 [1.89;3.97]	< 0.001
**TB contact**				
No	144 (42.99%)	191 (57.01%)	Ref	Ref
Yes	39 (67.24%)	19 (32.76%)	2.71 [1.52;4.98]	0.001
**HIV status**				
Negative	174 (49.86%)	175 (50.14%)	Ref	Ref
Positive	45 (49.45%)	46 (50.55%)	0.98 [0.62;1.56]	0.945
**Intravenous drug use**				
No	168 (49.70%)	170 (50.30%)	Ref	Ref
Yes	25 (49.02%)	26 (50.98%)	0.97 [0.54;1.76]	0.928
**Alcohol Abuse**				
No	152 (47.50%)	168 (52.50%)	Ref	Ref
Yes	39 (56.52%)	30 (43.48%)	1.43 [0.85;2.44]	0.178
**Drug resistance**				
MDR	212 (56.53%)	163 (43.47%)	Ref	Ref
XDR	42 (93.33%)	3 (6.67%)	10.2 [3.63;44.4]	< 0.001

**Figure 5:**
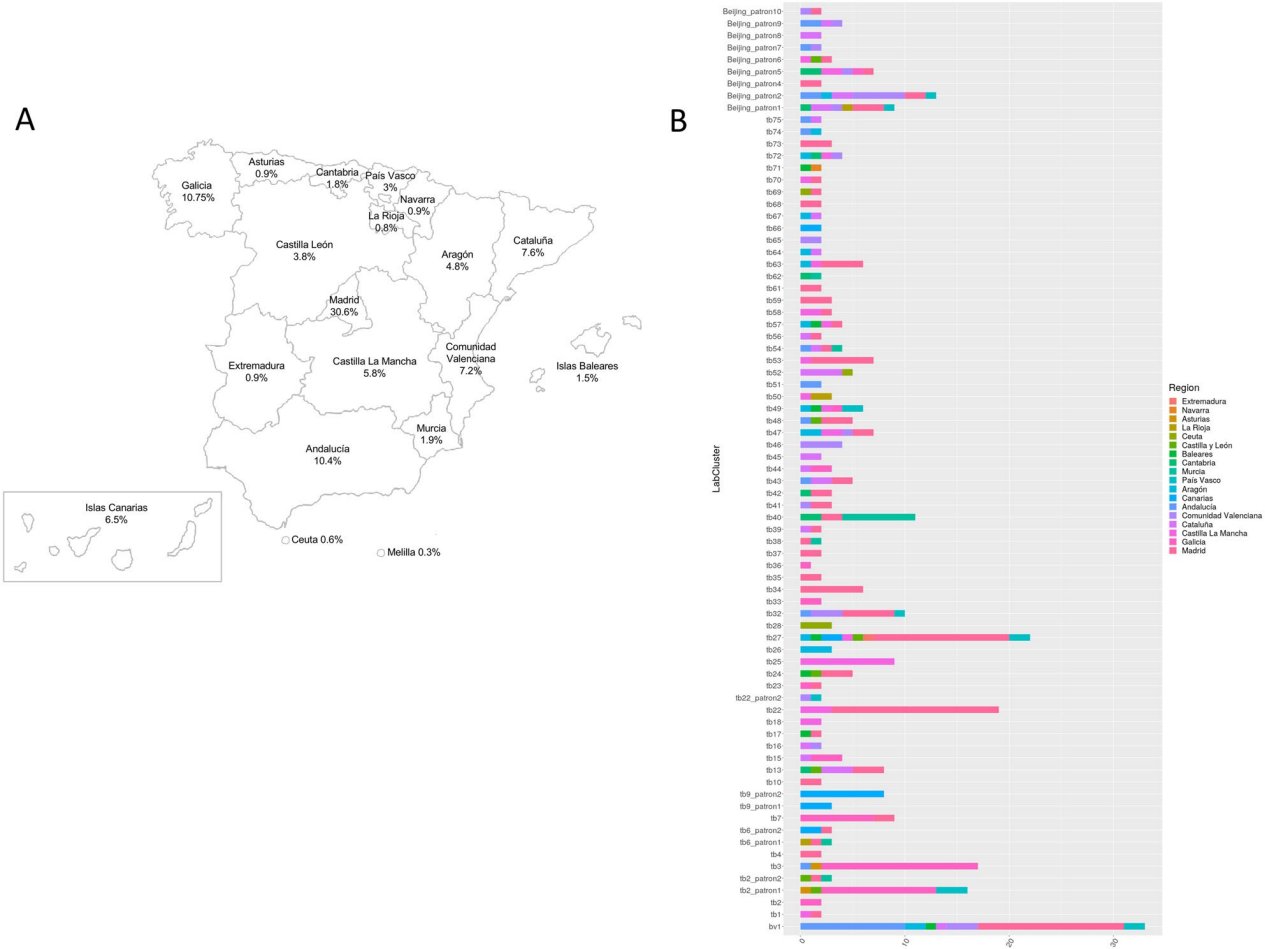
(**A**) Map of the regions or Autonomous Communities with the percentage of samples studied of each region. (**B**) Distribution of the clusters by geo-location of the isolates, the 77 clusters were classified in Spanish Regional-Clusters, if all the isolates were detected in one region, or Inter-Regional-Clusters, if involved cases coming from two or more different regions.

All regions, in which Spain is administratively divided, participated in our study by sending several samples to our laboratory (Fig. 5A). The geo-location of the isolates and the country of origin of the patients were investigated by clustering in order to clarify the circulation routes of the disease. According to the geo-location of the isolates, 77 clusters were classified in Spanish-Regional-Clusters (n = 26), if all the isolates were detected in one region, or in Inter-Regional-Clusters (n = 51), if the involved cases came from two or more regions (Fig. 5B). According to the country of origin of the cases, clusters were differentiated in Native-Cluster (n = 21) if grouped only Spaniard cases, Foreign-Clusters (n = 31) and Mixed-Clusters (n = 25), if both populations were included in a cluster. In order to know the impact of the importation of the MDR-TB in our country, we could observe that 19 of the Foreign-Clusters involved cases coming from a single country and that a foreign-born patient was the index case in 23 (71.9%) Mixed-Clusters. In a combined way, Cluster *tb27,* the most dispersed Interregional Cluster, geo-located in 8 different regions, was one of the largest Foreign Clusters, grouping 22 cases (Fig. 5B). Conversely, the largest cluster, *bv1*, was an Inter-Regional- and Native-Cluster, which grouped 33 isolates in seven different regions. Notably, 14 of the 19 Foreign-Clusters involving cases coming from the same country were Inter-Regional-Clusters: six from Romania, three from Peru, two from the Russian Federation and three more clusters whose patients came from Ethiopia, Moldavia and Ukraine, respectively. Since 2009, 252 genotyping data (31.9%) were integrated into the TB notification database, TESSy. Eighty-seven of these isolates, grouping from one to seven, participated in 20 European-clusters.

## Discussion

This work presents the results of the molecular study of the MDR isolates identified in Spain from 1998 to 2017. During this uninterrupted nationwide study, the molecular techniques and the recommendations for application have undergone important changes. The first standardised method used was IS*6110*-RFLP*,* which provides additional valuable information about this mobile element^10,11^. Afterwards, MIRU-VNTR was recommended, whose discrimination power and use in molecular epidemiology had been previously demonstrated^5,12–14^. In the last years, WGS has been applied to investigate outbreaks, and to analyse phylogeny and resistance^15–17^. In 2018, pilot studies carried out in Europe showed the added value of WGS for diagnosing TB as well as for detecting and tracing TB transmission events^2^. As result, WGS was proposed for surveillance in Europe, although standardization is still pending. Spain has incorporated this technology since 2018, combining with MIRU-VNTR to enable comparison with the previous obtained data. The global use of WGS will provide the possibility of developing other rapid methods, such us allele-specific oligonucleotide PCR, used in other studies to detect specific transmitted strains in subsequent years^18^.

According to the criteria fixed for our study, a single isolation of each case of MDR-TB was realized. For the 9.6% of the cases, two or more samples were isolated and received in different years of the study. This could represent a proxy indicator of the fail of treatment. These data are consistent with those reported in a recent meta-analysis (8% failure or relapse)^19^.

The genotyping analysis of our study showed a 48.4% of clustering, which represents a substantial increase with respect to the 33.8% and 32.7% obtained by the Spanish network in three and eleven year follow-up periods^4,5^. This high percentage would not be influenced by the presence of the largest cluster *bv1*, as the major number of cases was registered in the first two years and consequently they were included in the prior studies. Besides, we have documented that some clusters persisted throughout the period of time studied, indicating overall increase in clustering. Few studies have been carried out in other low-incidence settings at the national level in a similar time-frame. At the European level, 19% of clustering was found in Denmark over 15 years. Other shorter studies realized in Switzerland and Germany showed 24.4% and 49% of clustering, respectively, in seven years of follow-up^20^. On the other hand, a recent 4-year follow-up study in Portugal showed the highest clustering rate of 63.4%^21^. These differences in clustering and the persistence of some patterns should take us to reconsider the four-year time-frame as the most appropriate follow-up time to complement the molecular epidemiological studies^13^. The majority of clusters identified in our study belonged to L4. This is consistent with previous studies carried out in our geographical area^9,21^, however, the largest cluster was caused by an animal linage strain, coming from an existent outbreak during the nineties^22,23^.

The number of MDR-TB cases distributed along the period studied was not uniform. It was in the first year of study that the largest number, 12% of cases, was recorded. In contrast, the least number of cases was collected in the last year. It should be noted that the official sources showed a consistent trend in relation to MDR-TB data^2^. Otherwise, the country of origin of the cases studied reflects a parallelism with the phenomenon of immigration in Spain. In our study, the majority of cases were Spaniards until 2002, when foreign origin cases incremented. In the years following 2008, the decrease of cases observed can be explained by the "Great Recession" that affected Spain and led to an important departure of immigrants^24^. Likewise, most of the foreign-born cases came from the European Region and the Americas Region, and more specifically, from some of the highest-TB-burden countries in the EU/EEA, such as Romania^25^.

The descriptive analysis of the study population was in line with the classic facts for tuberculosis. The majority of our MDR-TB cases were male, although the M/F ratio was lower for Spanish pan-susceptible TB cases than those detected in EU/EEA^26^. The average age of the population studied was around 40 years old, close to the 38.3 years of average age described in a recent meta-analysis study of 12 030 patients^19^. In addition, the foreign-born population in Spain was younger than Spaniards. It is consistent with the profile of tuberculosis in Spain, with an average age of 51.2 years for native TB and 37.9 years for TB cases born abroad^27^. Considering the clinical characteristics of the disease, pulmonary TB, previous TB and TB contact were, as expected, the major forms of disease presentation. The excess in the percentage of XDR-TB cases (10.7%) could be explained by the outbreak that had affected mostly HIV positive individuals (cluster *bv1)* in the nineties caused by a well-characterized *M. bovis* strain^22,23^, with the last case in our study in 2014.

The analysis of the clusters according to the characteristic of the patients showed that the largest group, *bv1* cluster, was merely detected in Spaniards and mainly in the first two years of the study. Regarding the cluster duration, the most prolonged cluster was *Beijing pattern2* cluster, an Inter-Regional and Foreign-Cluster, whose index case was identified in 2000 and accumulated cases until 2017. Impressively, this *Beijing pattern2* cluster, the largest Beijing cluster identified in our study, accumulates 13 cases coming from five different East European countries and identified in 6 different regions. In addition to detecting foreign MDR-TB, our study supports the need for a more discriminative tool for Beijing isolates, which is in agreement with other studies that remark that MIRU-VNTR is not the most appropriate technique in this lineage^18^.

A higher proportion of foreign-born was found in cluster, in contrast with data obtained by other European countries^5,21,28^. Related to the Foreign-Clusters, MDR transmission could be occurring outside Spain. Most of these clusters were Inter-Regional-Clusters, what could be an indicative of importations of common strains from abroad. These patients could have arrived ill, or at least infected, to our country, what reports a high TB burden in their origin countries^28–30^. For instance, *tb27* cluster detected cases coming from Equatorial Guinea, most of these cases developed MDR-TB within 3 months after their arrival. Similarly, for *tb23*, which included two adopted girls from Ethiopia resided in different Spanish regions, was certainly verified^5,29^. Furthermore, the fact that three of these foreign-MDR genotypes were also present in TESSy, supports that they were likewise circulating in Europe outside our country. In contrast, the detection of 25 Mixed-Clusters makes us speculate the presence of transmission of MDR-TB in our own country.

The present study is subject to a number of limitations. For instance, the transfer of MDR-TB isolates was carried out voluntarily. Nevertheless, counting the participation of the national reference laboratory for mycobacteria and with the agreement of 90% of the mycobacterial laboratories of the National Health System, allowed us to consider that our results should not differ substantially from the real MDR-TB situation in Spain. Notably, all Spanish regions participated. Also, it has to be taken into account the considerable proportion of clinically diagnosed TB, with the consequent loss of microbiology confirmed cases (70.4%)^31^. Besides, it could happen that two identical genetic isolates analysed with two different techniques in two different times-frames, were not detected as being clonally related. Finally, the health information systems do not disaggregate TB outcome data. In this sense, information on contact tracing, treatment outcome and complete resistance profile was only available for a variable proportion of patients, so these relationships could not be explored. Due to the limited discriminatory power of RFLP and MIRU-VNTR, tracing transmission was not elucidated, however, our study shows an approximate picture of the MDR strains that circulated in our country between 1998 and 2017. Undoubtedly, WGS applied to future isolates would better track disease transmission than the present study.

In conclusion, the continuity of genotyping for 20 years, together with the overlapping of standard methods used, has allowed us to create a real portrait of the circulating MDR/XDR strains over 18 years in Spain. Having a networked system and a long study period is an advantage over partial and short-term molecular studies. The report and centralisation of the genotypes in the European Union provides more information about the MDR-TB situation in Spain by finding similar genotype in other countries. The use of WGS, as an exclusive tool for molecular typing, will facilitate better discrimination of MDR strains, although doing so without considering existing data will reduce the traceability of cases and may make it difficult to monitor MDR-TB.

## Data Availability

All genotype data used for this publication were stored in the University of Zaragoza and are available according to the Spanish national rules. Data from TESSy were provided by Spain and released by ECDC. The views and opinions of the authors expressed herein do not necessarily state or reflect those of ECDC. The accuracy of the authors’ statistical analysis and the findings they report are not the responsibility of ECDC. ECDC is not responsible for conclusions or opinions drawn from the data provided. ECDC is not responsible for the correctness of the data and for data management, data merging and data collation after provision of the data. ECDC shall not be held liable for improper or incorrect use of the data.
