# Author Correction: The value of the continuous genotyping of multi-drug resistant tuberculosis over 20 years in Spain

**DOI:** 10.1038/s41598-025-90681-1

**Published:** 2025-03-13

**Authors:** María José Iglesias, Daniel Ibarz, Alberto Cebollada, Jéssica Comín, María Soledad Jiménez, María C. Vázquez, Sofía Samper, The Spanish Working Group on MDRTB, T. Cabezas, A. Reyes, I. Ruiz, P. García, M. D. López, L. Cardeñoso, I. Jesús de la Calle, P. Ruiz, J. C. Alados, J. Román, R. Villa-Real, J. Saavedra, C. Amores, P. Bermúdez, M. A. Sánchez, N. Montiel, S. Bernal, J. A. Lepe, N. Batista, E. Roldán, L. Torres, C. Navarro, P. Chocarro, M. J. Aldea, J. Viñuelas, M. A. Vitoria, J. J. Palacios, H. Villar, P. Prendes, M. Blanco, F. Vázquez, M. Telenti, I. Sánchez, L. Carbo, S. Escobar, A. Ramírez, C. Gallegos, M. C. Pérez, M. Lecuona, O. Díez, R. Copado, I. Campos, F. Cañas, C. Salas, C. Fernández, M. P. Roíz, I. Barba, E. Manrique, R. Carranza, A. Sánchez Maroto, A. González, E. Rodríguez, V. Martino, C. Sánchez, C. Martínez, P. Robles, E. Simarro, C. Romero, R. López, M. D. Blanco, T. Nebreda, J. Rodríguez, J. M. Fernández, E. Álvarez, M. L. Jaime, M. D. Tejero, A. Alberte, E. Oteda, G. Megías, C. Labayru, R. Ibáñez, A. Campos, P. Carrero, J. M. Villó, T. Sans, I. Pujol, X. Clivillé, J. B. Castellví, J. de Batlle, D. Mariscal, C. Prat, M. García, F. Alcalde, C. Gallén, G. Sauca, E. Cuchi, C. Alonso, F. Corcoy, G. Schmidt, M. T. Tortola, E. Garduño, J. J. Moreno, P. Hernández, I. Montes, J. Roman, P. Alonso, A. Rodríguez, L. Barbeyto, B. Fernández, D. Domínguez, R. Villanueva, I. Iglesias, F. J. Vasallo, J. Sevillano, A. Pascual, M. García, M. L. Pérez del Molino, V. Martino, E. Ugalde, R. Dopereiro, J. A. Cuadros, I. Pelayo, J. Cacho, R. Cogollos, M. Páez, S. Prieto, R. Fernández, P. López, D. Domingo, R. Millán, I. Bonilla, P. Merino, C. Toro, M. J. Ruiz, M. Menéndez, P. Romero, M. Tato, M. Simón, A. Urmeneta, A. Delgado, L. García, J. Cobos, J. Merino, E. Aznar, J. Piqueras, M. D. Navarro, J. M. Artero, A. Navascués, A. Gil, J. Leiva, L. Elorduy, E. Urra, P. Idígoras, E. Pérez-Trallero, A. Canut, J. L. Barrios, L. Michans, R. Ayarza, F. García, M. J. Unzaga, M. Navarro, N. Gonzalo, C. Martín, C. Martínez, A. Gimeno, M. Elia, P. López, S. Sabater, J. C. Rodríguez, M. Santos, M. Bosque, J. López, E. Tabernero, M. I. Galán

**Affiliations:** 1 Universidad de Zaragoza, Zaragoza, Spain; 2 CIBER de Enfermedades Respiratorias, Madrid, Spain; 3 Instituto Aragonés de Ciencias de La Salud, Zaragoza, Spain; 4 Laboratorio de Investigación Molecular-UIT, IIS Aragón, Instituto Aragonés de Ciencias de La Salud, Hospital Universitario Miguel Servet, Pº Isabel la Católica 1-3, planta calle, CP 50009 Zaragoza, Aragón Spain; 5 Centro Nacional de Microbiología, Instituto de Salud Carlos III, Madrid, Spain; 6 Ministerio de Sanidad, Madrid, Spain; 7 H. Poniente, El Ejido, Almería, Spain; 8 H. Torrecárdenas, Almería, Spain; 9 H. Punta de Europa, Algeciras, Spain; 10 H. Univ. Puerta del Mar, Cádiz, Spain; 11 H. de Jerez de la Frontera, Cádiz, Spain; 12 H. Comarcal Puerto Real, Cádiz, Spain; 13 Hosp. Univ. de Puerto Real, Cadiz, Spain; 14 H. Reina Sofía, Córdoba, Spain; 15 H. Virgen de la Nieves, Granada, Spain; 16 Hosp. Univ. San Cecilio, Granada, Spain; 17 H. San Juan de la Cruz, Úbeda, Jaén, Spain; 18 H. Juan Ramón Jiménez, Huelva, Spain; 19 H. San Agustín, Linares, Spain; 20 Complejo H. Carlos Haya, Málaga, Spain; 21 H. Ntra. Sra. de la Victoria, Málaga, Spain; 22 H. Costa del Sol, Marbella, Spain; 23 H. Ntra. Sra. De Valme, Sevilla, Spain; 24 H. Virgen del Rocío, Seville, Spain; 25 H. Virgen de la Macarena, Sevilla, Spain; 26 H. La Merced, Osuna, Sevilla, Spain; 27 H. San Jorge, Huesca, Spain; 28 H. de Alcañiz, Teruel, Spain; 29 H. Obispo Polanco, Teruel, Spain; 30 H. Royo Villanova, Zaragoza, Spain; 31 H. Univ. Miguel Servet, Zaragoza, Spain; 32 H. Univ. Lozano Blesa, Zaragoza, Spain; 33 H. San Agustín, Avilés, Spain; 34 H. de Cabueñes, Gijón, Spain; 35 H. Monte Naranco, Oviedo, Spain; 36 H. Gral. de Asturias, Oviedo, Spain; 37 Instituto Nacional de Silicosis, Oviedo, Spain; 38 H. Verge del Toro, Mahon, Spain; 39 Policlínica Miramar, Palma de Mallorca, Spain; 40 H. Univ. Son Espases, Palma de Mallorca, Spain; 41 H. Joan March, Bunyola, Mallorca Spain; 42 H. Son Llatzer, Palma de Mallorca, Spain; 43 H. Univ. de Canarias, La Laguna, Spain; 44 H. Ntra. Sra. Candelaria, Sta. Cruz de Tenerife, Spain; 45 H. Gral. de Lanzarote, Madrid, Spain; 46 H. Doctor Negrín, Las Palmas, Spain; 47 H. Insular, Las Palmas, Spain; 48 H. Sta. Cruz, Liencres, Cantabria Spain; 49 H. Marqués de Valdecilla, Santander, Spain; 50 H. Sierrallana, Torrelavega, Spain; 51 H. Univ. de Ciudad Real, Ciudad Real, Spain; 52 H. Gutiérrez Ortega, Valdepeñas, Spain; 53 H. General La Mancha Centro, Alcázar de San Juan, Spain; 54 Hosp. Virgen de Altagracia, Manzanares, Spain; 55 H. Gral. de Guadalajara, Madrid, Spain; 56 H. Virgen de la Salud, Toledo, Spain; 57 H. Ntra. Sra. del Prado, Talavera de la Reina, Spain; 58 H. Virgen de la Luz, Cuenca, Spain; 59 H. Gral. de Albacete, Madrid, Spain; 60 H. de Hellín, Albacete, Spain; 61 H. del Bierzo, Ponferrada, Spain; 62 H. Monte San Isidro, León, Spain; 63 Complejo H. de León, León, Spain; 64 H. Virgen de la Concha, Zamora, Spain; 65 H. Clínico Univ. de Salamanca, Madrid, Spain; 66 H. Gral. Río Carrión, Palencia, Spain; 67 H. San Telmo, Palencia, Spain; 68 H. Clínico Univ. de Valladolid, Madrid, Spain; 69 H. Río Hortega, Valladolid, Spain; 70 H. Gral. Yagüe, Burgos, Spain; 71 H. Univ. de Burgos, Burgos, Spain; 72 H. Ntra. Sra. de Sonsoles, Ávila, Spain; 73 H. de Soria, Madrid, Spain; 74 H. de Segovia, Madrid, Spain; 75 H. Valls, Tarragona, Spain; 76 H. Mora de Ebro, Tarragona, Spain; 77 H. Sant Joan de Reus, Tarragona, Spain; 78 H. San Pau i Santa Tecla, Tarragona, Spain; 79 H. Doctor Trueta, Girona, Spain; 80 H. Parc Taulí, Sabadell, Barcelona, Spain; 81 H. Universitari Germans Trias i Pujol, Badalona, Barcelona, Spain; 82 H. Arnau de Vilanova, Lérida, Spain; 83 H. Univ. de Bellvitge, Hospitalet de Llobregat, Barcelona, Spain; 84 H. San Jaume, Calella, Barcelona, Spain; 85 Consorci Sanitari de Mataró, Barcelona, Spain; 86 H. Mutua de Terrassa, Barcelona, Spain; 87 H. de la Cruz Roja, Hospitalet de Llobregat, Barcelona, Spain; 88 H. San Camilo, San Pere de Ribes, Barcelona, Spain; 89 H. de Terrasa, Barcelona, Spain; 90 Hospital Universitari Vall d’Hebron, Barcelona, Spain; 91 H. Univ. Infanta Cristina, Badajoz, Spain; 92 H. de Mérida, Badajoz, Spain; 93 H. Ciudad de Coria, Cáceres, Spain; 94 H. Virgen del Puerto, Plasencia, Spain; 95 H. de Monforte, Lugo, Spain; 96 H. Xeral de Calde, Lugo, Spain; 97 H. Cristal Piñor, Orense, Spain; 98 H. Santa Maria Nai, Orense, Spain; 99 H. Arquitecto Marcide, Ferrol, Spain; 100 H. Juan Canalejo, Coruña, Spain; 101 H. Xeral-Cíes, Vigo, Spain; 102 H. Meixoeiro, Vigo, Spain; 103 Clínica Povisa, Vigo, Spain; 104 H. Montecelo, Pontevedra, Spain; 105 H. Provincial, Pontevedra, Spain; 106 Complejo Univ. de Santiago de Compostela, Madrid, Spain; 107 H. de Conxo, Santiago de Compostela, Spain; 108 H. San Pedro, La Rioja, Spain; 109 H. Gral. de la Rioja, Logroño, Spain; 110 H. Príncipes de Asturias, Alcalá de Henares, Spain; 111 H. Fuenfría, Cercedilla, Spain; 112 H. Univ. de Getafe, Madrid, Spain; 113 H. de Móstoles, Madrid, Spain; 114 H. Severo Ochoa, Leganés, Madrid, Spain; 115 H. de Fuenlabrada, Madrid, Spain; 116 Fundación Jiménez Díaz, Madrid, Spain; 117 H. Doce de Octubre, Madrid, Spain; 118 H. de la Princesa, Madrid, Spain; 119 H. Puerta de Hierro, Madrid, Spain; 120 H. San Carlos, Madrid, Spain; 121 H. Carlos III, Madrid, Spain; 122 H. Gregorio Marañón, Madrid, Spain; 123 H. Infantil Univ. Niño Jesús, Madrid, Spain; 124 H. La Paz, Madrid, Spain; 125 H. Ramón y Cajal, Madrid, Spain; 126 H. Central de la Defensa Gómez Ulla, Madrid, Spain; 127 H. Enfermedades del Tórax, Cantoblanco, Madrid, Spain; 128 H. Univ. Fundación de Alcorcón, Alcorcón, Spain; 129 Hosp. de El Escorial, Madrid, Spain; 130 MEGALAB, Madrid, Spain; 131 UNILABS, Madrid, Spain; 132 Laboratorio BR Salud. Laboratorio Central de Madrid, San Sebastián de los Reyes, Spain; 133 H. Sta. María del Rosell, Cartagena, Spain; 134 H. Morales Messeguer, Murcia, Spain; 135 H. Reina Sofía, Murcia, Spain; 136 Complejo hospitalario de Navarra, Pamplona, Spain; 137 Clínica Universitaria de Navarra, Pamplona, Spain; 138 H. San Eloy, Baracaldo, Spain; 139 H. de Cruces, Baracaldo, Spain; 140 Complejo H. Donostia, San Sebastián, Spain; 141 H. Santiago Apóstol, Vitoria, Spain; 142 H. Txagorritxu, Vitoria, Spain; 143 H. de Galdakao, Vizcaya, Spain; 144 H. Sta. Marina, Bilbao, Spain; 145 H. de Basurto, Bilbao, Spain; 146 H. Vega Baja, Orihuela, Alicante, Spain; 147 H. Sant Joan, Alicante, Spain; 148 H. de Villajoyosa, Alicante, Spain; 149 H. Gral. Univ, Alicante, Spain; 150 H. Virgen de la Salud, Elda, Spain; 151 H. Verge dels Lliris, Alcoy, Spain; 152 H. Gral.de Castellón, Madrid, Spain; 153 H. Gral. de Elche, Madrid, Spain; 154 H. la Fe, Valencia, Spain; 155 H. Arnau de Vilanova, Valencia, Spain; 156 H. de la Cruz Roja, Ceuta, Spain; 157 H. de Melilla, Melilla, Spain

Correction to: *Scientific Reports* 10.1038/s41598-020-77249-x, published online 24 November 2020

The Funding section in the original version of this Article was incomplete.

The molecular methods applied in this work were supported by the Carlos III Health Institute in the context of two consecutive Grants FIS15/0317 and 18/0336 and JC was awarded a scholarship by the Government of Aragon/European Social Fund, “Building Europe from Aragon”.

Now reads:

The molecular methods applied in this work were supported by the Carlos III Health Institute (ISCIII) in the context of two consecutive Grants FIS15/0317 and 18/0336, co-financed by European Regional Development Funds of the European Commission: “A way of making Europe”. JC was awarded a scholarship by the Government of Aragon/European Social Fund, “Building Europe from Aragon”.

The original Article has been corrected.

